# Virtual Waiting Room: The New Narrative of Waiting in Oncology Care

**DOI:** 10.1007/s13187-024-02496-9

**Published:** 2024-09-02

**Authors:** Miroslav Světlák

**Affiliations:** 1 Department of Medical Psychology and Psychosomatics, Faculty of Medicine, Masaryk University, Brno, Czechia; 2 Department of Comprehensive Cancer Care, Masaryk Memorial Cancer Institute, Brno, Czechia

**Keywords:** Virtual waiting room, Circular entry model, Patient engagement, Caregiver support, Oncology communication

## Abstract

This conceptual study introduces the “virtual waiting room,” an innovative, interactive, web-based platform designed to enhance the waiting experience in oncology by providing personalized, educational, and supportive content. Central to our study is the implementation of the circular entry model, which allows for non-linear navigation of health information, empowering patients to access content based on their immediate needs and interests. This approach respects the individual journeys of patients, acknowledging the diverse pathways through which they seek understanding and manage their health. The virtual waiting room is designed not only to support patients but also to facilitate stronger communication and shared understanding between patients, caregivers, and families. By providing a shared digital space, the platform enables caregivers and family members to access the same information and resources, thereby promoting transparency and collective knowledge. This shared access is crucial in managing the emotional complexities of oncology care, where effective communication can significantly impact treatment outcomes and patient well-being. Furthermore, the study explores how the circular entry model within the virtual waiting room can enhance patient autonomy and engagement by offering customized interactions based on user feedback and preferences. This personalized approach aims to reduce anxiety, improve health literacy, and prepare patients more effectively for clinical interactions. By transforming passive waiting into active engagement, the virtual waiting room turns waiting time into a meaningful, informative period that supports both the psychological and informational needs of patients and their support networks.

## The Hospital Waiting Room: A Crucial Component of Outpatient Care

The hospital waiting room is an inevitable component of outpatient care. Despite hospitals’ best efforts, patients spend much time there on their treatment and recovery journey. Patients face many emotions in this environment, anticipating both positive and negative medical updates and challenging physical examinations [1]. This period significantly influences their personal lives and psychological well-being, bringing back memories of past treatments and exposing them to the suffering of others in the shared waiting room space [2]. Every visit to the oncologist carries inherent traumatic potential, making it a pivotal and emotionally charged event in the treatment trajectory. Waiting can often lead to increased frustration, anger, fear, and anxiety, which can, in turn, intensify the perception of physical pain and negative psychological symptoms [3]. Stress in the waiting room also naturally impacts patient behavior, potentially negatively influencing patient-physician interaction [4]. Many patients desire to leave the hospital as soon as possible without any unnecessary delays [5].

## Waiting Room as Part of the Treatment: A New Narrative

While waiting may seem like something to be endured, it is also a valuable opportunity to promote patient’s mental and physical health. It is a space where healthcare experts can engage positively with patients, helping them change their waiting room experience, increase their medical literacy, offer relevant information, strengthen responsibility for their health and self-care, modulate their emotional state before seeing the doctor [6], and introduce them to practices for mental health support. This aligns with the findings of Li and colleagues [7], which documented that patients and caregivers primarily seek detailed and understandable information about the diagnosis, treatment options, side effects, and prognosis, alongside emotional support to navigate the psychological impacts of cancer. The American Society of Clinical Oncology (ASCO) Consensus Guideline on Patient-Clinician Communication [8] emphasizes the importance of effective communication in oncology to enhance the patient-clinician relationship, patient and clinician well-being, and family well-being. Recommendations focus on establishing goals for conversations, exploring patient and family information needs, fostering trust, providing information tailored to patient preferences, responding empathically to emotions, discussing prognosis and treatment options including clinical trials and end-of-life care, facilitating family involvement, overcoming barriers to communication, and discussing care costs.

Despite the availability of information, many patients still experience a significant gap between the information provided and their actual understanding or needs. This discrepancy often arises from factors such as health literacy, where patients with lower literacy may find it challenging to comprehend medical terminology and the implications of different treatment options [9]. Communication barriers also play a role, as limited consultation time and the lack of clear, concise information tailored to a patient’s understanding level can impede effective communication. Additionally, the psychological distress accompanying a cancer diagnosis can affect a patient’s ability to process information and make informed decisions. Patient preferences add another layer of complexity; while some patients may want detailed information about every aspect of their treatment, others might feel overwhelmed by too much information and prefer just the essentials.

These challenges underscore the need to develop and implement innovative communication and educational tools that allow patients to maximize and efficiently use the available time with the physician while offering alternative sources of information and support. These tools and resources, including digital applications, online health portals, educational materials, and community forums, offer patients the ability to access information at the time and in the manner that best suits their individual needs and circumstances.

## Current Practices in the Field

Hospitals are adopting a wide array of evidence-based solutions and best practices to enhance patient experiences and communication, tailored through various channels to engage and improve patient well-being during the waiting. This includes disseminating information via digital and physical media, such as televisions and informational displays [4], leaflets, and roll-ups [6], alongside the strategic use of auditory stimuli through background music [10] and nature sounds [11]. Visual stimuli also play a significant role, with the integration of comedic content on television [12], scenic visualizations of nature and landscapes [13], and visual art [14] to cultivate a positive and healing waiting space. An increasing number of studies indicate that thoughtful design of waiting areas can significantly enhance both the subjective comfort and perceived quality of healthcare environments [15]. Furthermore, many hospitals have expanded their physical resources by integrating digital tools, particularly through the creation of informational websites and patient web portals, as documented by Coughlin et al. [9]. These online platforms frequently include educational sections that offer patients and visitors convenient access to health information and resources, effectively extending the supportive hospital environment into the digital realm. Additionally, hospitals are increasingly utilizing social media platforms like Facebook and Instagram to disseminate a variety of news and updates, further enhancing patient engagement and communication.

## Bringing It All Together: The Missing Piece of the Puzzle

While the form and content of communication channels in waiting rooms are robust and comprehensive, there is a notable gap in the overarching framework guiding their utilization—a unifying concept that could transform these individual elements into a cohesive, systematic, and personalized approach to support patients’ mental and physical health more effectively. A lot of useful information is usually dispersed in different places within hospitals. Some are in leaflets handed out by doctors and nurses, some are on the hospital’s website, some are in external resources that experts recommend, and some run in TV spots in waiting rooms.

In the digital era, patients also encounter an overwhelming abundance of health-related information online, varying significantly in quality and accuracy. This underscores the critical importance of ensuring that the information patients access supports their treatment and adheres to standards set by relevant professional societies and hospitals. It is imperative to guide patients toward reliable sources that align with our/their treatment plans.

In this context, an approach that not only simplifies the process of finding relevant information but also enhances the patient’s sense of control and engagement with their treatment is essential. This goes beyond passive exposure to methods like music, art, or nature pictures. Personalized and evidence-informed medicine [16] does not seek a one-size-fits-all method but asks for whom, when, and at what stage of the disease a particular method is most effective. Research into mental health promotion offers numerous effective methods [17]. The challenge is not what to use but how to use it and in what narrative and conceptual framework to incorporate existing methods and approaches. Accepting the fact that most people in hospital waiting rooms are mentally healthy [18] even if they are in a zone of psychological discomfort and exposed to chronic stress, good education and scientifically proven methods can have a significant impact on a large proportion of them. Those who meet the diagnostic criteria for a psychopathology disorder may also be referred for further specialized care.

## Virtual Waiting Room: The Next Step Beyond the Patient Portals in Hospital Ecosystems

One innovative solution and potential pillar of personalized care through technology could be the virtual waiting room (VWR). This interactive, web-based platform could integrate current knowledge in health literacy development, cutting-edge medical education, and mental health support, accessible to patients at their hospital. This approach not only makes health education more accessible but also ensures it is delivered at a critical moment—while patients are waiting to see their doctor. This timing can make patients more receptive to information that aids their understanding, alleviates anxieties, and empowers them to participate actively in their care. Additionally, the information and actions conveyed through the VWR are endorsed by the hospital and its experts, thereby enhancing adherence to its content and objectives.

Unlike physical waiting rooms, constrained by space, layout, and hygiene standards, the virtual space offers an infinite range of possibilities that can ultimately enhance the physical space by improving how people feel and interpret their waiting experience.

The VWR is not just a digital extension of the physical waiting area; it represents a paradigm shift in patient interaction with healthcare environments before consultations. It provides relevant information effectively for health support, increases health literacy, and boosts self-support capacity. The VWR can also serve as a resource hub for social and financial support, as well as patient advocacy groups.

Moreover, the concept of a virtual waiting room extends beyond patient management, offering potential as a communicative bridge for oncology patients and their family members or caregivers. By providing a platform where both parties can observe and understand the full spectrum of discussions, treatment options, and ongoing healthcare topics, a virtual waiting room can significantly enhance and facilitate their communication. This not only fosters better dialogue between patients and doctors but also encourages family members to engage more deeply with the patient’s care process. The VWR is also a valuable platform for hospitalized patients, ensuring continuous access to support and information.

## Meaningful Waiting

To fully appreciate its potential, let’s explore the virtual waiting room (VWR) concept in a structured manner. Imagine you have arrived at our hospital and are sitting in the waiting area. We will guide you step by step through the virtual experience, ensuring a clear and informative journey.

### Virtual Waiting Room: Come In

The VWR can be introduced through short TV spots, leaflets, and roll-ups with links to the website or QR codes on the seats. It can be recommended by medical doctors, nurses, psychologists, and volunteers. The VWR can be presented with a unifying motto or with categories referring to specific solutions (Table 1).

**Table 1:** Motto and landing topic examples

Motto	Category
Waiting can be a meaningful part of your treatment	How to prepare for a consultation with a doctor
Wait meaningfully, visit our virtual waiting room	How to go through the treatment
Virtual waiting room is bigger than just the room you sit in: let us start, where you are now	I need to calm down

### Login Options

In the VWR, patients are offered various levels of access, each providing a different degree of personalization and integration with the healthcare system:

- Anonymous access: patients and caregivers can enter the waiting room without any form of login, allowing them to freely explore all content.
- Email-based login: for those who prefer a tailored experience without sharing personal medical information, logging in with an email address offers continuity across visits. This feature enables patients to track their screening results over time, access a history of previously viewed content, discover new materials, and see what is trending among other users.
- Personalized access with ID: by registering with their personal ID number, patients can link their virtual waiting room experience directly to their medical records within the hospital system. This approach allows for highly personalized content, including educational materials specific to the patient’s diagnosis, disease stage, and upcoming treatments. Screenings and feedback are integrated into the patient’s medical records for comprehensive care coordination with their physician. For example, a patient undergoing chemotherapy might receive links to videos on managing side effects, while someone in remission could access content on wellness and survivorship. This personalization extends to language preferences, literacy levels, and cultural considerations, ensuring that all patients find the resources accessible and engaging.
- Shared access: this registration option enables patients and their caregivers to create linked accounts, allowing them to view each other’s activity within the platform. By seeing what topics and content each other is engaging with, they can better communicate about the patient’s concerns and the caregiver’s perspectives. This shared visibility promotes more open discussions regarding treatment and care, ensuring that both parties are informed and aligned in their approach to managing the illness. This can facilitate a deeper understanding and support system, enhancing both the patient’s and caregiver’s ability to navigate the complexities of the healthcare journey together.

### Personalization: Let Us Start Where Our Patients Are

**Fig. 1:**
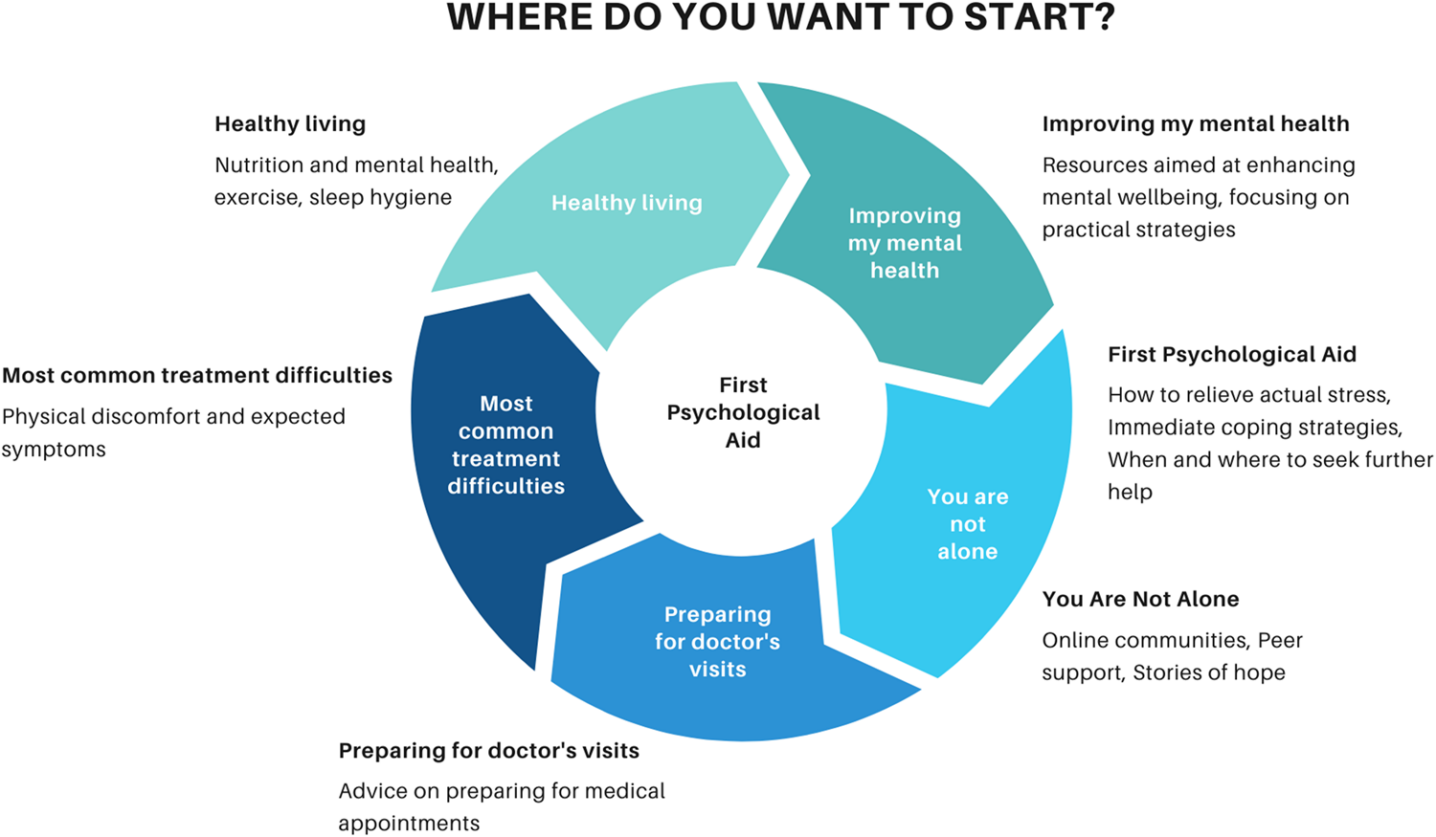
The landing web page of VWR: the example of the circular entry model

The key to the VWR’s effectiveness lies in its user-friendly interface, designed to meet patients where they are and address their current needs through simple, intuitive categories. We propose employing the circular entry model (CEM) for the VWR, an innovative approach currently being trialed in the national health information portal of the Czech Republic [19]. Effective portals align with patient preferences and are easy to navigate, enhancing self-directed use without extensive training [20–22]. This model abandons the traditional hierarchical structure, instead allowing patients to navigate content based on their immediate needs, interests, and preferences. By facilitating a non-linear exploration of topics, the platform respects the unique experiences of everyone, recognizing that the journey to understanding and managing health can vary greatly from person to person. An example of a model landing page is presented in Fig. 1.

The themes in the VWR are interrelated, allowing patients to navigate through their immediate concerns and explore connected topics. For instance, an oncology patient might start with a theme on the fear of disease relapse. A short video featuring a clinical psychologist or physician can address this fear, acknowledge it using principles of first psychological aid, and explain emotion regulation strategies, such as acceptance. This might then lead the patient to related topics like the relationship between disease relapse and lifestyle choices or secondary prevention strategies.

Hospitals can customize their content to meet their specific needs and preferences. However, clinical experience and research suggest a consistent list of topics that patients are interested in (see Table 2). Research by Catania et al. [2] involving 355 oncology patients highlights the emotional impact of waiting rooms. Key findings reveal that 83% of patients experience significant emotional distress while waiting. Many patients (35%) are upset by discussing their condition with others, and 26% are emotionally impacted by seeing other sick individuals. Patients suggested improvements such as organizing alternative activities like professional meetings, doctor consultations, and fun activities like music therapy. A notable preference was for the ability to leave the waiting room, using messaging systems to alert them when their turn approaches, to mitigate feelings of lost freedom or time. This study underscores the need for a waiting room environment that addresses the complex emotional needs of cancer patients, transforming waiting time into a more constructive and less distressing experience.

**Table 2:** An example of a sequential, trauma-sensitive approach for addressing fear in cancer recovery

Steps	Type of content	Name	Description
Initial overview	Headline 1-min video	It is “OK” to feel fear sometimes	Fear is a natural part of recovery: learning to understand and work with it can enhance your well-being
Understanding	Short article or 5-min video	Understanding fear of recurrence	This content explores why fear of recurrence arises, providing both statistical and psychological insights
What to do	Interactive content choice	Coping strategies or survivor stories or medical insights	Everyone requires a unique approach to manage and confront the fear
I need more	Lecture or podcast (30 min)	Series of survivor interviews or emotion regulation strategies with clinical psychologist	Let us explore the evidence-based approaches available
Further resources	Links and PDFs	Provides additional materials for further exploration	Links to support groups, books on managing illness anxiety, and professional services

By selecting a category that reflects their immediate concern or curiosity, patients can quickly access resources specifically tailored to their needs. This approach goes beyond passive exposure to methods like music, art, or nature pictures that hospitals might provide without personalization. By allowing patients to define their greatest need at the moment, the VWR empowers them to take an active role in their healthcare experience. As a trauma-sensitive method, this approach carefully tailors exposure to the patient’s readiness and consent, while transparently outlining the full spectrum of possibilities and pathways they may consider [23].

This empowerment is crucial in oncology, where patients often face a loss of control over their treatment and its impact on their quality of life. Providing tools to better understand their condition, manage their mental health, and stay informed about new research and treatment options can significantly enhance their overall wellbeing and treatment outcomes.

### Trauma-Sensitive Dosing of Information

It is essential to recognize that our objective is not to forge the perfect patient—one who is well-informed, makes sound decisions, embraces their illness, and employs methods to support both mental and physical well-being, while feeling completely at ease with their circumstances. Instead, our aim is to create an environment that facilitates this process, yet we acknowledge that it may not be suitable for everyone. Our approach is designed to honor individual differences, recognizing that each person’s path to health is distinct. By providing a system that is both flexible and adaptable, we strive to meet a broad spectrum of needs and preferences, ensuring that our platform is valuable to everyone, no matter their stage in the health journey. Rather than prescribing a uniform solution, we offer an array of tools and resources, empowering individuals to select those that best fit their unique situations and needs. To optimize patient understanding and engagement, it is imperative to present information in concise, manageable segments. Employing a graduated approach—from broad overviews to detailed specifics—allows patients to customize their consumption of information according to their individual needs and emotional capacities (see Table 2).

This method is particularly crucial within a trauma-sensitive framework [23], where respecting and adapting to the patient’s emotional and cognitive states is essential. Furthermore, avoiding the delivery of overly lengthy documents or complex technical videos is important. Instead, information should be introduced gradually. This approach not only upholds their autonomy but also empowers them, significantly enhancing their ability to manage their healthcare journey (Table 3).

**Table 3:** The list of clinically relevant topics for the virtual waiting room

Category	Subcategories	Description
Understanding my treatment	Medication guides, side effects of the treatment, therapy techniques, recovery expectations	Detailed explanations of treatments and procedures, tailored to specific health conditions or therapy types
Managing stress	Mindfulness and meditation, breathing exercises, physical activities	Stress management techniques, including articles, videos, and interactive tools
Latest research	Innovations in medicine, clinical trials, emerging therapies	Updates on medical research findings and implications for treatment
Improving my mental health	Self-care strategies, building resilience	Resources aimed at enhancing mental wellbeing, focusing on practical strategies
eHealth tools for your health	Applications for mental and physical health support	Map of available applications verified by our expert team
Anxiety management	Identifying triggers, reduction techniques, professional help	Resources for understanding and managing anxiety, emphasizing self-help and professional guidance
Healthy living	Nutrition and mental health, exercise, sleep hygiene	Wellness topics contributing to physical and mental health
Connecting with others	Support groups, patient advocacy groups, family resources	Engagement with support networks through various channels
Understanding your rights	Privacy and confidentiality, medical records, advocacy	Education on patient rights within the healthcare system
Financial resources and assistance	Insurance coverage, social support and charity programs, managing costs	Options to help with financial or social problems during and after treatment
Common sources of discomfort	Physical discomfort, emotional challenges, navigating uncertainty	Strategies for relief and understanding of various discomforts
You are not alone	Online communities, peer support, stories of hope	Importance of community and shared experiences, resources to connect and find support
Preparing for doctor’s visits	Checklist for appointments, what to bring, questions to ask	Advice on preparing for medical appointments, ensuring patients are ready and informed
First psychological aid	How to relieve actual stress, recognizing distress, immediate coping strategies, when to seek further help	Techniques for immediate psychological support and guidance on when professional help is needed
How I am doing: screening	Types of screenings, what results mean	It is important to have standardized feedback on how we are doing and to have comparisons over time
How to read scientific information and assess its reliability	Understanding research studies, evaluating sources, identifying bias, and disinformation	Resources to help understand and critically evaluate scientific research and its sources for reliability and bias
Alternative medicine and integrative medicine in oncology	How to understand alternative methods in oncology and how to think about their effect	Evidence-based alternative methods and integrative medicine

## E-Health: Mapping Your Path to Wellness

In today’s digital age, eHealth applications for mental and physical health care are increasingly accessible. The global expenditure on mobile mental health solutions is expected to approach US$500 million in 2022, with a projected annual growth rate of 20% [24]. This estimate may be conservative, given the sector’s rapid increase from US$203 million to US$269 million—a 32% rise—from 2019 to 2020.

However, the expertise and scientific evidence supporting these applications vary significantly. Most people cannot easily determine which app is optimal for their specific situation and stage of illness. Developing more eHealth applications is economically unsustainable for health systems when numerous solutions already exist. Instead, our task is to curate a meaningful list and create a mental map for our patients, incorporating existing eHealth solutions with our expert recommendations. We aim to guide patients on when, why, and how to use these applications effectively.

This initiative can be a valuable component of a VWR in any medical field. The safety and effectiveness of eHealth solutions can be guaranteed by hospital experts or national medical professional societies. A similar map is being developed at the European level [25].

Creating a platform for eHealth applications that recommends apps based on individual needs and expert advice has great potential to improve healthcare access. This approach simplifies the process of finding and selecting relevant health apps, increasing the likelihood that users will use the most appropriate and effective tools. Developing such an eHealth map represents a significant step toward personalized, informed healthcare accessible from the comfort of users’ homes. By integrating expert assessments, user reviews, and advanced filters, this platform can significantly enhance user health and wellbeing.

## The Research Potential of VWR: From Free Browsing to Digital Phenotyping and Personalized Content

Despite the increasing adoption of patient portals, their usage remains modest among cancer patients, and research on their effectiveness is still in its early stages [9]. While 90% of healthcare systems in the United States now offer patient portals to access electronic health records (EHRs), only 15 to 30% of patients utilize these platforms [26]. To enhance usage, it is essential to understand what motivates individuals to engage with such tools. By comprehending patient motivations, healthcare providers can better tailor their approaches to meet patient needs and promote the widespread adoption of these valuable resources. It is evident that the effectiveness of patient portals is linked to the context in which they are used, with integrated health service networks demonstrating more positive outcomes [26].

Patient portals often lack the wide range of features available in a VWR. The research potential of the VWR platform is vast and multifaceted. By implementing this platform, healthcare providers can gather invaluable data on patient needs in real time. This data can reveal patterns related to patients’ digital behavior—known as their “digital phenotype”—including their interactions with digital health resources, content preferences, and engagement timing and duration [27]. These insights are pivotal for tailoring health interventions and educational content to better meet patient needs and enhance care quality. Additionally, the platform can serve as a living laboratory for testing new forms of digital health communication and intervention, evolving continuously based on user feedback and emerging healthcare needs.

For research purposes, it is useful to initially categorize users as either patients receiving treatment or family members. This distinction helps explore the specific information needs and communication preferences of these groups, illuminating how the virtual waiting room can meet their distinct needs.

## Real-Time Feedback and Psycho-Oncology Screening

Integrating regular screenings for stress levels, wellbeing, and quality of life assessments into the platform can provide immediate, actionable insights to healthcare providers. Routine distress screening in oncology emphasizes the critical importance of recognizing and addressing distress in cancer patients beyond mental health specialists. There is a consensus among many organizations on the need to prioritize identifying distress and its sources as part of a comprehensive assessment of a patient’s wellbeing, advocating for routine distress screening in cancer care. Screening is not a diagnostic tool but a means to initiate conversations, assess severity, and identify those needing further evaluation. Despite developing and validating screening tools, translating screening into tangible patient benefits depends on the availability, offering, and acceptance of evidence-based treatments [28].

In the VWR context, individuals can receive feedback on their performance regarding specific health variables. This feedback is comparative, offering insights relative to a research-based reference group, a hospital-specific reference group, and tracking individual progress over time. This option extends to viewing these comparative groups. The VWR focuses on identifying significant health concerns, such as quality of life, anxiety, depression, psychological distress, and treatment-associated symptoms [29]. This feature is instrumental in enabling timely healthcare interventions and fostering tailored care plans. For example, relaxation videos within the VWR can help participants measure their emotional state before and after viewing, allowing the system to recommend the most effective strategies for managing anxiety, stress, or worry [5]. Furthermore, the VWR facilitates personalized feedback and guidance on self-help techniques or discussions with healthcare providers. Clinically significant findings could be integrated into medical records, accessible by doctors, enhancing the continuity and personalization of care.

## Social Support and Community Building

Integrating a chat feature for sharing experiences and mutual support introduces a social dimension to the VWR. This virtual community space can significantly enhance the patient experience by providing emotional support, reducing feelings of isolation, and fostering a sense of belonging among individuals facing similar health challenges. The chat functionality within the VWR can facilitate the formation of thematic groups where individuals freely exchange perspectives, experiences, and coping strategies. Such interactions are instrumental in creating a support network that extends beyond traditional patient-care provider dynamics. All interactions on the platform, particularly those within the community chat feature, will prioritize user privacy and confidentiality, adhering to strict data protection standards.

## New Possibilities for Communicating with Patients and Their Families

The findings of a recent systematic review [30] about the impact of Health Information Technology (HIT) on communication between cancer patients and their healthcare providers demonstrate that technology improves access to care, supports patient knowledge and shared understanding, enhances therapeutic alliances, leads to higher quality medical decisions, enhances patients’ ability to manage emotions, improves family and social support, and enhances patients’ empowerment and agency. Effective use of technology can increase patient satisfaction, enable better decision-making, and strengthen the patient-provider relationship. The study concludes that technology-based solutions can significantly bolster the well-being of cancer patients, assist healthcare providers in making better-informed decisions, and improve the overall therapeutic alliance between patients and doctors.

In addition, the large language models (LLMs) are poised to significantly reshape the future of psycho-oncology healthcare, integrating increasingly into various eHealth solutions across oncology [31]. The evolving question is not whether LLMs will be used, but rather how extensively they will be implemented and in which specific contexts they will provide the most benefit. AI’s ability to process vast amounts of data through natural language processing and machine learning significantly improves the screening and identification of oncology patients’ psychosocial needs, facilitating timely mental health referrals and support. AI assistants offer significant potential in supporting patients by answering their inquiries, organizing their documentation, and elucidating aspects of their treatment. This technology can enhance the patient experience by providing immediate, reliable information and structured guidance tailored to individual health needs [32]. AI can also accurately predict the need for mental health referrals, underscoring its potential as an integral part of eHealth solutions for supporting the mental health of oncology patients. This method enhances early detection and referral, effectively addressing the frequently unmet psychosocial needs of these patients and providing a new tool for effective psycho-oncology screening of stress-related symptoms. Through its use, AI assistants become pivotal in improving patient care by integrating advanced technology into routine clinical practice [33].

Although still in the early stages, artificial intelligence, particularly through LLMs, is poised to play also a significant role in advancing psychotherapy [34]. AI assistants could become an integral part of virtual waiting rooms, providing short-term support psychotherapy and mental health assistance. Similarly, they could enhance patient education by utilizing scanned or electronically uploaded patient documentation to tailor educational content specifically to individual needs.

## Understanding Limits and Obstacles for Realistic Implementation of VWR

In assessing the efficacy and reach of VWR, it is essential to acknowledge their limitations, as they do not universally cater to all demographic groups. Research indicates that older individuals often engage less with these technologies, suggesting significant barriers in technology adoption linked to age. The success of virtual waiting rooms also heavily relies on patients’ access to technology, health literacy, and motivation to participate actively in managing their health [9].

Moreover, the feasibility of implementing these technologies varies globally, largely depending on regional healthcare infrastructure and available funding. Even with internet access provided within hospital settings, assuming that all individuals have consistent access to high-speed internet outside these settings is flawed, affecting patient engagement continuity.

It is critical to note that virtual waiting rooms are not intended to replace direct interactions with healthcare professionals but to complement them, offering a platform that enhances traditional care. They serve as a potential discussion point between doctors and patients, enabling the integration of health screening results directly into medical records, which can inform more meaningful health discussions during patient visits. Overall, while virtual waiting rooms represent a significant advancement in healthcare technology, their adoption and impact are limited by various demographic, technological, motivational, economic, and infrastructural factors.

## Virtual Waiting Room: It Is Not About Time, It Is About Approach

In the healthcare sector, the concept of the VWR is evolving from a futuristic idea to a practical solution aimed at enhancing patient care. One of the most common refrains in medicine is the perceived lack of time for implementing new systems or practices. However, when we delve deeper into the daily activities of doctors, nurses, and psychologists—their interactions with patients, the advice they dispense, and the recommendations they make—it becomes evident that the content for a virtual waiting room has long been in the making.

Our experience across medicine shows that patients frequently ask where they can find and recall information, but we typically lack a comprehensive source to direct them to. We usually hand out a leaflet or refer them to the hospital website, but the information is often sketchy and incomplete. Moreover, there is no consensus among doctors and other health professionals on what to recommend, especially when they cannot refer to a single and guaranteed source of information.

The real challenge lies not in creating content from scratch but in reorganizing and repurposing the wealth of existing information into accessible formats like videos, podcasts, texts, and guided recommendations. This shift in perspective highlights that the bottleneck is often our approach rather than actual time constraints.

The primary goal of the VWR is to streamline the flow of information, making it readily accessible at different stages of patient care. This ensures that patients are directed to reliable resources, crafted and curated by healthcare professionals, that are consistent with the treatment standards and philosophies particular to their needs. By changing our approach to how this content is assembled and presented, we can significantly enhance efficiency and ensure that every patient receives the same foundational knowledge, tailored to their specific journey through treatment.

This strategy not only saves time but also reinforces the quality of patient education, ensuring that all patients have access to trusted, professional guidance whenever they need it. This reimagined approach to the virtual waiting room could transform patient waiting time into an informative, empowering experience that supports their treatment pathway.

## Conclusion

The introduction of the VWR dynamically linked to the broader digital ecosystem of a hospital represents a forward-thinking approach to patient care. By offering personalized, accessible, and timely health education and mental health support, the VWR has the potential to transform waiting time into a valuable opportunity for patient empowerment and engagement. This innovative platform stands to make a significant impact on the patient experience, offering a model for how healthcare providers can leverage digital technologies to meet the complex needs of their patients more effectively.

The proposed CEM for the VWR is an innovative approach that prioritizes flexibility, personalization, trauma-sensitive context, and interconnectedness in patient education and support. This model eschews a hierarchical structure, instead allowing patients to navigate content based on their immediate needs, interests, and personal health journey. By facilitating a non-linear exploration of topics, the platform respects the unique experiences of everyone, acknowledging that the path to understanding and managing health can vary widely from one person to another.

The VWR and its content could also act as a springboard for meaningful discussions about mental health between doctors and patients. By facilitating these conversations, we can enhance communication, build trust, and ultimately improve patient outcomes.

## Data Availability

This article is a theoretical study and does not involve any new datasets. All information regarding the concepts, frameworks, and analyses presented herein is contained within this publication. No additional data are available.

## References

[1] La Verde NM et al (2009) Perspective evaluation of emotional state of oncological patients in the waiting rooms. J Clin Oncol 27(15_suppl):e20693. 10.1200/JCO.2009.27.15_SUPPL.E20693

[2] Catania C et al (2011) ‘Waiting and the waiting room: how do you experience them?’ Emotional implications and suggestions from patients with cancer. J Cancer Educ 26(2):388–394. 10.1007/s13187-010-0057-2 20204572 10.1007/s13187-010-0057-2

[3] Lamba N, Niemierko A, Martinez R, Leland P, Shih HA (2020) The interaction of waiting time and patient experience during radiation therapy: a survey of patients from a tertiary cancer center. J Med Imaging Radiat Sci 51(1):40–46. 10.1016/J.JMIR.2019.08.008 31839482 10.1016/j.jmir.2019.08.008

[4] Fryburg DA (2021) What’s playing in your waiting room? Patient and provider stress and the impact of waiting room media. J Patient Exp 8. 10.1177/23743735211049880 10.1177/23743735211049880 PMC8641118 34869835

[5] Halámek F et al (2024) Enhancing patient well-being in oncology waiting rooms: a pilot field experiment on the emotional impact of virtual forest therapy. Front Psychol 15. 10.3389/fpsyg.2024.1392397 10.3389/fpsyg.2024.1392397 PMC11117429 38800677

[6] Světlák M, Šnajdrová V, Fialová K (2023) How patients can help us be even better doctors - educational leaflet ‘Before I go to the doctor’. Klin Onkol 37(4):307–313. Available from: https://pubmed.ncbi.nlm.nih.gov/38195385/38195385

[7] Li J, Luo X, Cao Q, Lin Y, Xu Y, Li Q (2020) Communication needs of cancer patients and/or caregivers: a critical literature review. J Oncol. 10.1155/2020/7432849 32454826 10.1155/2020/7432849 PMC7229568

[8] Gilligan T et al (2017) Patient-clinician communication: American Society of Clinical Oncology Consensus Guideline. J Clin Oncol 35:3618–3632. 10.1200/JCO 28892432 10.1200/JCO.2017.75.2311

[9] Coughlin S (2018) A review of web portal use by oncology patients. J Cancer Treat Diagn 2(6):1–6. 10.29245/2578-2967/2018/6.1154 10.29245/2578-2967/2018/6.1154 PMC6342494 30680374

[10] Lai JCY, Amaladoss N (2022) Music in waiting rooms: a literature review. Health Environ Res Design J 15(2):347–354. 10.1177/19375867211067542 10.1177/19375867211067542 PMC9072951 34961338

[11] Watts G, Khan A, Pheasant R (2016) Influence of soundscape and interior design on anxiety and perceived tranquillity of patients in a healthcare setting. Appl Acoust 104:135–141. 10.1016/j.apacoust.2015.11.007

[12] Genç H, Saritas S (2020) The effects of watching comedy videos on anxiety and vital signs in surgical oncology patients. Explore 16(6):401–406. 10.1016/j.explore.2020.02.009 32247709 10.1016/j.explore.2020.02.009

[13] Reese G, Stahlberg J, Menzel C (2022) Digital shinrin-yoku: do nature experiences in virtual reality reduce stress and increase well-being as strongly as similar experiences in a physical forest? Virtual Real 26(3):1245–1255. 10.1007/s10055-022-00631-9

[14] Nanda U, Chanaud C, Nelson M, Zhu X, Bajema R, Jansen BH (2012) Impact of visual art on patient behavior in the emergency department waiting room. J Emerg Med 43(1):172–181. 10.1016/j.jemermed.2011.06.138 22325555 10.1016/j.jemermed.2011.06.138

[15] Bazley C, Vink P, Montgomery J, Hedge A (2016) Interior effects on comfort in healthcare waiting areas. Work 54(4):791–806. 10.3233/WOR-162347 27447409 10.3233/WOR-162347

[16] Attia P, Gifford B (2023) Outlive: the science & art of longevity, 1st edn. Harmony, New York

[17] Breitbart WS, Butow PN, Jacobsen PB, Lam WWT, Lazenby M, Loscalzo MJ (eds) (2023) Psycho-oncology, 4th edn. Oxford University Press, Oxford

[18] Grassi L et al (2023) Anxiety and depression in adult cancer patients: ESMO Clinical Practice Guideline. ESMO Open 8(2). 10.1016/j.esmoop.2023.101155 10.1016/j.esmoop.2023.101155 PMC10163167 37087199

[19] Světlák M, Suchý A, Slezáčková A, Šumec R, Cacková H, Malatincová T, Kerberová M, Soukupová M, Komenda M (2024) Guide to mental health. National health information portal [Internet]. Prague: Ministry of Health of the Czech Republic and Institute of Health Information and Statistics of the Czech Republic [cited 2024 May 22]. Available from: https://www.nzip.cz/dusevni-zdravi

[20] Irizarry T, Shoemake J, Nilsen ML, Czaja S, Beach S, DeVito Dabbs A (2017) Patient portals as a tool for health care engagement: a mixed-method study of older adults with varying levels of health literacy and prior patient portal use. J Med Internet Res 19(3). 10.2196/JMIR.7099 10.2196/jmir.7099 PMC5391436 28360022

[21] Portz JD et al (2019) Using the technology acceptance model to explore user experience, intent to use, and use behavior of a patient portal among older adults with multiple chronic conditions: descriptive qualitative study. J Med Internet Res 21(4). 10.2196/11604 10.2196/11604 PMC6475817 30958272

[22] Osborn CY, Mayberry LS, Wallston KA, Johnson KB, Elasy TA (2013) Understanding patient portal use: implications for medication management. J Med Internet Res 15(7). 10.2196/jmir.2589 10.2196/jmir.2589 PMC3713921 23823974

[23] Davidson CA, Kennedy K, Jackson KT (2023) Trauma-informed approaches in the context of cancer care in Canada and the United States: a scoping review. Trauma Violence Abuse 24(5):2983–2996. 10.1177/15248380221120836 36086877 10.1177/15248380221120836 PMC10594848

[24] Deloitte (2024) Mental health app market [Internet]. Deloitte Touche Tohmatsu Limited. Available from: https://www2.deloitte.com/xe/en/insights/industry/technology/technology-media-and-telecom-predictions/2022/mental-health-app-market.html

[25] European Commission (2018) eHealth Hub Platform - the map of the European digital health ecosystem [Internet]. Available from: https://digital-strategy.ec.europa.eu/en/news/ehealth-hub-platform-map-european-digital-health-ecosystem

[26] Lyles CR, Nelson EC, Frampton S, Dykes PC, Cemballi AG, Sarkar U (2020) Using electronic health record portals to improve patient engagement: research priorities and best practices. Ann Intern Med 172(11):S123–S129. 10.7326/M19-0876 32479176 10.7326/M19-0876 PMC7800164

[27] Huckvale K, Venkatesh S, Christensen H (2019) Toward clinical digital phenotyping: a timely opportunity to consider purpose, quality, and safety. NPJ Digital Med 2(1) Nat Publ Group 10.1038/s41746-019-0166-1 10.1038/s41746-019-0166-1 PMC6731256 31508498

[28] Mitchell AJ (2021) Screening and assessment for distress. In: Breitbart WS, Butow PN, Jacobsen PB, Lam WWT, Lazenby M, Loscalzo MJ (eds) Psycho-oncology, 4th edn. Oxford University Press, Oxford, pp 121–130

[29] Hui D, Bruera E (2017) The Edmonton Symptom Assessment System 25 years later: past, present, and future developments. J Pain Symptom Manage 53(3):630–643. 10.1016/J.JPAINSYMMAN.2016.10.370 28042071 10.1016/j.jpainsymman.2016.10.370 PMC5337174

[30] ElKefi S, Asan O (2021) How technology impacts communication between cancer patients and their health care providers: a systematic literature review. Int J Med Inform 149 Elsevier Ireland Ltd 10.1016/j.ijmedinf.2021.104430 10.1016/j.ijmedinf.2021.104430 PMC8131252 33684711

[31] O’Connor S et al (2024) The application and use of artificial intelligence in cancer nursing: a systematic review. Eur J Oncol Nurs 68:102510. 10.1016/j.ejon.2024.102510 10.1016/j.ejon.2024.102510 38310664

[32] Kurian M, Adashek JJ, West H (2024) Cancer care in the era of artificial intelligence. JAMA Oncol 10(5):683–683. 10.1001/JAMAONCOL.2023.7263 38546590 10.1001/jamaoncol.2023.7263

[33] Nunez JJ, Leung B, Ho C, Ng RT, Bates AT (2024) Predicting which patients with cancer will see a psychiatrist or counsellor from their initial oncology consultation document using natural language processing. Commun Med 4(1). 10.1038/s43856-024-00495-x 10.1038/s43856-024-00495-x PMC11001970 38589545

[34] Stade EC et al (2024) Large language models could change the future of behavioral healthcare: a proposal for responsible development and evaluation. NPJ Mental Health Res 3(1). 10.1038/s44184-024-00056-z 10.1038/s44184-024-00056-z PMC10987499 38609507

